# Complete Genome Sequence of Seneca Valley Virus CH-01-2015 Identified in China

**DOI:** 10.1128/genomeA.01509-15

**Published:** 2016-01-21

**Authors:** Qiwen Wu, Xiaoya Zhao, Yanshan Chen, Xiaoming He, Guanqun Zhang, Jingyun Ma

**Affiliations:** a College of Animal Science, South China Agricultural University, Guangzhou, Guangdong, People’s Republic of China; b Guangdong Wen’s Foodstuff Group Co., Ltd., Xinxing, Guangdong, People’s Republic of China

## Abstract

The complete genome sequence of Seneca Valley virus (SVV), a single-stranded RNA virus that causes porcine vesicular disease in China, has been sequenced and analyzed. This Chinese isolate shares 94.4 to 97.1% sequence identity to another 8 strains from Canada, Brazil, and the United States. This is the first report of SVV infecting swine in China.

## GENOME ANNOUNCEMENT

Seneca Valley virus (SVV), which causes porcine idiopathic vesicular disease, was first identified in either contaminated fetal bovine serum or porcine trypsin during the cultivation of PER.C6 cells (1) and isolated from pigs displaying suspicious vesicular disease when pigs were imported to the United States from Canada in 2007 (2, 3). Recently, it has also been identified in Canada and Brazil (4, 5). SVV is a nonenveloped single-stranded RNA virus, which belongs to the genus *Senecavirus* and family *Picornaviridae*, and it is most closely related to members of the *Cardiovirus* genus (6, 7).

In the summer of 2015, pigs in the swine farms in Guangdong Province exhibited vesicular disease, including ulcers in the nasal and oral cavities, anorexia, lameness, and acute death of newborn piglets. To determine the causative agent, pooled samples were tested and were negative for foot-and-mouth disease, swine vesicular disease, and vesicular stomatitis, but they were positive for SVV. Genome RNA was extracted from the samples using the AxyPrep body fluid viral DNA/RNA miniprep kit (Axygen, USA), according to the manufacturer’s instructions. The complete genome of SVV CH-01-2015 was generated with reverse transcriptase PCR (RT-PCR) using 7 pairs of primers amplifying 7 overlapped fragments. The primers were designed using the Primer 5 based on the sequence of SVV-001 (GenBank accession no. NC_011349.1). The 7 amplified RT-PCR products were purified and cloned into pMD19-T and sent to Genewiz, Inc. for sequencing.

The verified complete genome for the Chinese isolate SVV CH-01-2015 comprises 7,285 nucleotides (GenBank accession no. KT321458), excluding a 3′-poly(A) tail. The genome contained an open reading frame (ORF) encoding a 2,181-amino acid (aa) polyprotein, a 668-nucleotide (nt) 5′ untranslated region (UTR), and a 71-nt 3′ UTR. The SVV CH-01-2015 complete genome sequence shares 94.4 to 97.1% nucleotide identity with eight complete SVV genomes in GenBank, and its polyprotein shares 98.4 to 99.1% amino acid sequence identity with the polyproteins of the eight SVV strains. To our knowledge, the identification of a Seneca Valley virus isolate (SVV CH-01-2015) in China was the first report of SVV in China. Furthermore, additional analysis to investigate the biological and molecular properties of SVV is necessary.

### Nucleotide sequence accession number. 

The genome sequence of SVV CH-01-2015 has been submitted to GenBank under the accession number KT321458.
